# *De novo *characterization of the gametophyte transcriptome in bracken fern, *Pteridium aquilinum*

**DOI:** 10.1186/1471-2164-12-99

**Published:** 2011-02-08

**Authors:** Joshua P Der, Michael S Barker, Norman J Wickett, Claude W dePamphilis, Paul G Wolf

**Affiliations:** 1 Department of Biology and Center for Integrated Biosystems, Utah State University, Logan, UT 84322-5305, USA; 2 Department of Ecology and Evolutionary Biology, University of Arizona, Tuscon, AZ 85721, USA; 3 Department of Biology, Institute of Molecular Evolutionary Genetics, and The Huck Institutes of the Life Sciences, The Pennsylvania State University, University Park, PA 16802, USA

## Abstract

**Background:**

Because of their phylogenetic position and unique characteristics of their biology and life cycle, ferns represent an important lineage for studying the evolution of land plants. Large and complex genomes in ferns combined with the absence of economically important species have been a barrier to the development of genomic resources. However, high throughput sequencing technologies are now being widely applied to non-model species. We leveraged the Roche 454 GS-FLX Titanium pyrosequencing platform in sequencing the gametophyte transcriptome of bracken fern (*Pteridium aquilinum*) to develop genomic resources for evolutionary studies.

**Results:**

681,722 quality and adapter trimmed reads totaling 254 Mbp were assembled *de novo *into 56,256 unique sequences (i.e. unigenes) with a mean length of 547.2 bp and a total assembly size of 30.8 Mbp with an average read-depth coverage of 7.0×. We estimate that 87% of the complete transcriptome has been sequenced and that all transcripts have been tagged. 61.8% of the unigenes had blastx hits in the NCBI nr protein database, representing 22,596 unique best hits. The longest open reading frame in 52.2% of the unigenes had positive domain matches in InterProScan searches. We assigned 46.2% of the unigenes with a GO functional annotation and 16.0% with an enzyme code annotation. Enzyme codes were used to retrieve and color KEGG pathway maps. A comparative genomics approach revealed a substantial proportion of genes expressed in bracken gametophytes to be shared across the genomes of *Arabidopsis*, *Selaginella *and *Physcomitrella*, and identified a substantial number of potentially novel fern genes. By comparing the list of *Arabidopsis *genes identified by blast with a list of gametophyte-specific *Arabidopsis *genes taken from the literature, we identified a set of potentially conserved gametophyte specific genes. We screened unigenes for repetitive sequences to identify 548 potentially-amplifiable simple sequence repeat loci and 689 expressed transposable elements.

**Conclusions:**

This study is the first comprehensive transcriptome analysis for a fern and represents an important scientific resource for comparative evolutionary and functional genomics studies in land plants. We demonstrate the utility of high-throughput sequencing of a normalized cDNA library for *de novo *transcriptome characterization and gene discovery in a non-model plant.

## Background

As the sister lineage to seed plants, ferns represent a critical clade for comparative evolutionary studies in land plants [1,2]. In contrast to seed plants, ferns typically retain the ancestral condition for a suite of life history traits (e.g. the lack of secondary growth, homospory, motile sperm, and independent free-living gametophyte and sporophyte generations). Ferns are thus an important outgroup for studying the evolution of wood, seeds, pollen, flowers, and fruit among other economically important characteristics found in seed plants, as well as the evolution of development in these complex structures and the expansion of gene families associated with seed plant evolution (e.g. transcription associated proteins). For reasons not yet fully understood, ferns typically have much higher chromosome numbers and larger genomes than seed plants [1,3,4].

Understanding the factors that influence these differences and their evolutionary consequences will require developing genomic resources in ferns to provide the necessary comparative context to understand the evolution of these traits [3-5]. Additionally, because ferns have evolved and maintained free-living and photosynthetic gametophyte and sporophyte life stages, they are an ideal group for studies of life-cycle evolution in land plants and genome function in independent haploid and diploid phases.

Among the genomic tools available for non-model organisms, expressed sequence tags (ESTs) have proven to be a rapid and cost effective strategy to develop sequence markers for comparative evolutionary and functional studies. While taxonomic sampling of plants in genome-scale projects has expanded substantially with dramatic decreases in sequencing cost, and increases in throughput, the development of genomic resources in ferns has lagged far behind those of other plant groups. This deficit has primarily been attributed to technical and economic barriers associated with the complex and large genomes in ferns, but is compounded by the limited agronomic value of most ferns [4]. To date (December 2010), genomic information in ferns is limited to a genetic linkage map [6] and a modest EST data set comprised of about 5,000 Sanger cDNA sequences [7] for *Ceratopteris richardii*, just over 30,500 ESTs for *Adiantum capillus-veneris *[8], and over 2,600 ESTs in *Osmunda lancea *[9]. Fewer than 500 ESTs for *Pteridium aquilinum *have previously been sequenced and deposited in Genbank [9].

With the introduction of cost efficient and massively-parallel high-throughput sequencing technologies, genome-scale studies in non-model organisms are being actively pursued for gene discovery, expression profiling, SNP and SSR marker development, and studies in functional, comparative, and evolutionary genomics in taxa where little or no previous genomic information exists [10-27]. We chose the Roche 454 GS-FLX Titanium pyrosequencing technology to sequence a full length enriched normalized cDNA library for the gametophyte generation of the bracken fern, *Pteridium aquilinum *(L.) Kuhn.

*Pteridium *(family: Dennstaedtiaceae) is a cosmopolitan fern genus comprised of several closely related species that are well differentiated from other genera in the family. *Pteridium aquilinum *is the most widespread of the bracken species and is distributed throughout the northern hemisphere and Africa [28]. Bracken is notorious as a weed in open fields and is toxic to people and livestock. Despite its toxicity, bracken is eaten as a delicacy in several parts of the world, and due to its often high local abundance and large coarse stature, is sometimes used as thatching or packing material. Because bracken is common, easily cultured and manipulated, and can have a major economic impact, it has become one of the most intensively studied fern species.

Bracken has been used as a model system for the study of the fern life cycle [29-37], gametophyte development and the pheromonal mechanism of sex determination [38-45], cyanogenesis [46], carcinogenesis [47-49], invasion ecology [50-52], and climate change [53]. *Pteridium aquilinum *has a diploid chromosome count of 2n = 104 and a total genome size of about 9.8 Gbp [54].

This study was conceived to develop an extensive expressed gene sequence resource in ferns for evolutionary and functional genomics. We present the first comprehensive transcriptome characterization for a fern gametophyte, including an assessment of transcriptome coverage, gene family and functional representation, SSR identification, and a comparative analysis of gene sets across land plants.

## Results

### Sequencing and *de novo *assembly

**Figure 1:**
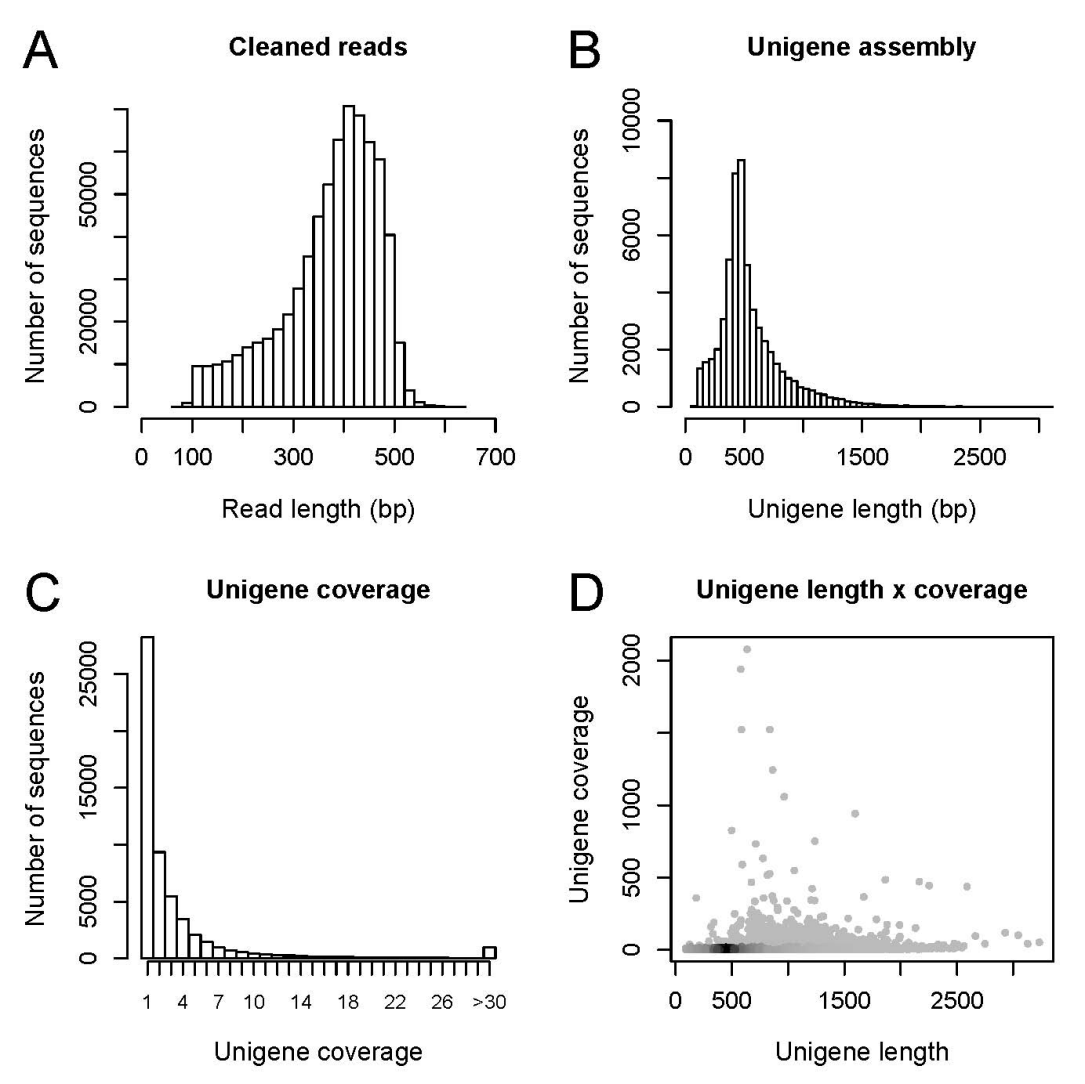
**Overview of *P. aquilinum *transcriptome sequencing and assembly**. (A) A histogram of the filter passed and adapter/quality trimmed Roche 454 GS-FLX Titanium read lengths. (B) A histogram of unigene lengths for the final unigene set after the 2-step assembly. Note that the longest unigene is 4,489 bp and the x-axis has been truncated at 3 kb. (C) A histogram of the average read-depth coverage for unigenes. The steep decline in coverage observed here is typical of normalized libraries [15]. Coverage values between 30× and 1800× have been binned (see the vertical axis in Figure 1D). (D) A density scatterplot showing the relationship between unigene length and coverage. Points with a higher local density are darker.

Raw Roche 454 GS-FLX Titanium reads were quality and adapter trimmed and size selected to yield 681,722 cleaned reads with a mean length of 372.6 bp and 254 Mbp of total sequence data (Table 1, Figure 1A). Reads were first assembled in MIRA v3.0rc4 [55] and the resulting assembly was passed through a second assembly step in CAP3 [56] to join additional contigs (Table 2). The resulting final assembly consisted of 56,256 unique sequences (i.e. retained singletons plus primary and secondary contigs, hereafter referred to as unigenes; Additional file 1). Unigenes had a mean length of 547.2 bp and summed to a total assembly length of 30.79 Mbp (Table 1, Figure 1B). The average read-depth coverage for the final unigene assembly was 7.0× (Table 2). The distribution of unigene coverage was highly left-skewed toward low coverage with an extremely long tail (maximum coverage was 2,078×; Figure 1C, D). The steep decline in read-depth coverage suggests that cDNA normalization was effective and is typical for a normalized library [15].

**Table 1:** Sequence statistics

	Raw reads	Cleaned reads	Unigenes
Number of sequences	730,577	681,722	56,256
Mean length (bp)	363.55	372.60	547.23
Standard deviation on length (bp)	118.60	96.36	276.06
Mode length (bp)	416	383	466
Median length (bp)	398	394	479
Range in length (bp)	2 - 624	78 - 624	86 - 3229
Total length (Mbp)	265.60	254.01	30.79

Summary statistics for sequence data at different stages of processing.

**Table 2:** Assembly summary statistics

	Primary assembly (MIRA)	Secondary assembly (MIRA+CAP3)
Number of reads assembled into contigs	574,134	640,285
Number of reads discarded during assembly	31,723	0
Number of 454 reads retained as singletons	75,865	9,714
Number of primary contigs (MIRA)	91,100	24,775
Number of secondary contigs (CAP3)	0	21,767
Total number of unique sequences (unigenes)	166,965	56,256
Mean unigene length (bp)	423.11	547.23
Largest unigene length (bp)	1,746	3,229
Total assembly length (Mbp)	70.65	30.79
Mean read depth coverage	3.03	6.96

A comparison of the primary and secondary assemblies. Secondary assembly was used to condense and join contigs and singletons from the primary assembly to reduce sequence level redundancy in the final unigene set.

### Transcriptome coverage and data quality

Because information about the actual size and composition of the transcriptome is often unknown, we utilized a simulation-based tool, ESTcalc [57], to estimate the expected depth and breadth of transcriptome coverage for this data set. The model for transcriptome coverage backing ESTcalc was parameterized using the well-characterized *Arabidopsis thaliana *transcriptome and several "next-generation" sequencing runs using normalized and non-normalized cDNA libraries [57]. Using the results from these simulations (retrieved using ESTcalc), our dataset is expected to cover 87% of the nucleotide positions in the transcriptome (Table 3), with every gene represented by at least one read (i.e. percent of genes tagged).

**Table 3:** Transcriptome coverage estimates: ESTcalc

Input Parameters	ESTcalc estimate	Actual
Number of technologies	1	1
Technology	454 GS-FLX	454 GS-FLX (Titanium)
Library type	normalized	normalized
MB/Plate	254	254.0076
Reads/Plate	681,722	681,722
BP/Read (mean)	372.6	372.6

**Predicted assembly statistics**		

Total Assembled Sequence (MB)	26.2	30.97
Unigene count	32,044	56,256
Mean unigene length (bp)	819	547.23
Mean unigene length (longest unigene per gene, bp)	1,143	--
Singleton yield (%)	19	17.2400
Percent transcriptome (%)	87	--
Percent of genes tagged (%)	100	--
Percent of genes with 90% coverage (%)	69.8	--
Percent of genes with 90% coverage by largest unigene (%)	56.4	--
Percent of genes with 100% coverage (%)	23.7	--
Percent of genes with 100% coverage by largest unigene (%)	22.2	--

Estimates of transcriptome coverage based on simulations modeled using the *Arabidopsis thalliana *floral transcriptome [57].

**Figure 2:**
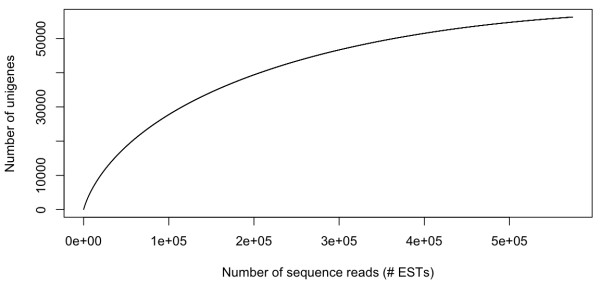
**Unigene accumulation curve**. The mean number of unigenes detected as a function of the number of reads sampled. The complete set of reads in the 2-step assembly were shuffled and drawn at random for 1,000 bootstrap replicates.

Additionally, 70% of the genes are predicted to be sequenced to 90% of their length. Consistent with these estimates, we were able to identify 333 of 357 (93.3%) *Arabidopsis *genes that are conserved as single copy genes across all Eukaryotes (i.e. ultra-conserved orthologs; UCOs [58]). Similarly, we detected 754 of 959 (78.6%) shared single copy tribes from *Arabidopsis thaliana*, *Populus trichocarpa*, *Vitis vinifera*, and *Oryza sativa *in our classification of unigenes in the PlantTribes database [59,60]). These two gene sets (UCOs and shared single copy tribes) represent a highly conserved subset of genes expected to be present in eukaryotic and plant genomes, respectively, and can be used as a proxy for gene detection and sampling breadth. As a final measure of gene detection in this data set, we utilized a bootstrapped data resampling approach using the distribution of reads in our final assembly (see methods section) to generate a unigene accumulation curve which plots the number of unigenes detected as a function of sequencing effort (Figure 2). Using this method, we estimate that on average 90%, 95% and 99% of the unigenes were tagged after approximately 378,683; 455,145; and 543,727 reads were sampled (Figure 2). On average, it took 59 reads to detect each of the last ten unigenes.

To identify potential contaminant sequences in the sample or sequencing library, we examined the taxonomic distribution of blastx hits for each unigene searched in the NCBI nr database. We examined both the taxonomic classification of the best hit as well as the lowest common ancestor (LCA) assignment for each unigene using MEGAN v.3.7.2 [61]. 34,740 unigenes had a positive a blast hit, of which only 1.8% had a best hit to an organism outside of the green plants and 1.1% received an LCA assigned taxon which is not within, or a super set of land plants (Table 4). We also examined the unigene set for potential genomic DNA contamination by screening unigenes for blastn hits to the complete chloroplast genome sequence of *Pteridium aquilinum *(HM535629 [62]). None of the chloroplast sequences identified in the transcriptome were longer than 3.5 kb or contained more than five adjacent genes (most spanned only a single gene) and thus can reasonably be considered putative transcripts [63,64]. That we did not detect any long fragments of chloroplast DNA in the transcriptome assembly gives us confidence that our DNase treatment during RNA extraction was effective and the resulting cDNA library used in sequencing is free of contaminant genomic DNA.

**Table 4:** Taxonomic distribution of unigene blastx hits in the nr database

	Best blastx hit		Lowest common ancestor for blastx hits	
**Taxonomic category**	**Number of unigenes**	**Percent of unigenes with hits**	**Number of unigenes**	**Percent of Unigenes with hits**

Eukaryotes	33,776	97.2%	32,059	92.3%
Green plants	33,406	96.2%	31,373	90.3%
"Green algae"	175	0.5%	78	0.2%
Land plants	33,231	95.7%	30,822	88.7%
"Bryophytes"	394	1.1%	2,197	6.3%
Vascular plants	32,837	94.5%	16,731	48.2%
Lycophytes	74	0.2%	13	0.0%
Ferns	928	2.7%	435	1.3%
Seed plants	31,835	91.6%	16,015	46.1%
Gymnosperms	8,000	23.0%	866	2.5%
Angiosperms	23,835	68.6%	10,572	30.4%
Animals	288	0.8%	63	0.2%
Fungi	0	0.0%	4	0.0%
Other eukaryotes	77	0.2%	12	0.0%
Bacteria	22	0.1%	91	0.3%
Artificial sequences, hits don't pass threshold, or taxon not assigned	20	0.1%	216	0.6%

Unigenes were searched in the NCBI nr protien database using blastx with an e-value threshold of 1e-10, keeping the best ten hits. Of the 56,256 unigenes, 34,740 (61.8%) had a positive hit. The lowest common ancestor (LCA) assignment for a sequence was calculated using the LCA algorithm implemented in MEGAN v3.9 [61] based on at least three blastx hits with a bitscore greater than 75 and within 10% of the best bitscore. Note: the predicted proteins from *Selaginella moellendorffii *are not currently included in the nr database and thus are not reflected in these results.

### Functional annotation

**Figure 3:**
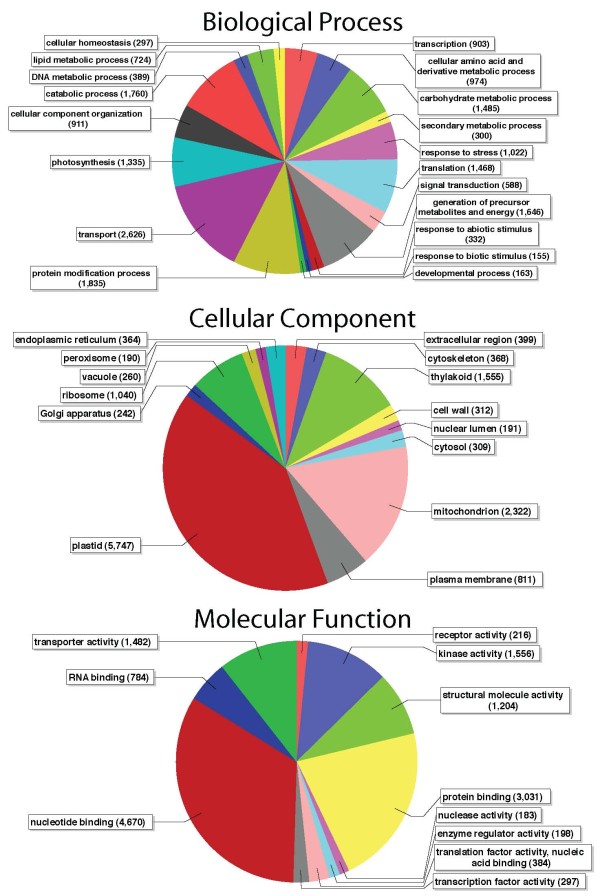
**Distribution of plant GO-slim functional categories**. The relative proportion of plant GO-slim terms represented by more than 150 unigenes for the three major categories in the GO vocabulary (biological process, cellular component, and molecular function).

Unigenes were annotated with gene ontology (GO) terms, enzyme codes, and conserved protein domain functions using the Blast2GO suite [65-67]. Unigenes were first interrogated against the NCBI nr protein database using a blastx e-value threshold of 1e-10, keeping the top 10 high scoring alignments, resulting in 34,740 unigenes (61.8%) with positive blast hits. The best blastx hits for these unigenes corresponded to 22,596 unique protein accessions in the nr database. The longest open reading frame (uncorrected six-frame translations automated in Blast2GO) from 29,357 unigenes (52.2%) had positive matches to conserved protein domains using InterProScan (IPS) searches implemented in Blast2GO. These results (nr blastx and IPS) were used to assign 87,137 GO terms to 25,999 unigenes (Additional file 2). These GO terms were used to map 11,243 enzyme codes to 8,993 unigenes. Enzyme codes were then used then to retrieve and color 144 KEGG pathway maps. To assess whether the frequency of functional categories present in the *Pteridium *transcriptome differ significantly from the suite of gene functions present in other plants, we compared the GO terms assigned to *Pteridium *unigenes with the GO classification for all genes in the *Arabidopsis thalliana *genome (TAIR9 GO annotation downloaded on 13 September 2010) using a two-tailed FDR-corrected Fisher's exact test. When using the full GO classification, none of the GO terms in *Pteridium *are significantly enriched or underrepresented relative to the full GO classification for *Arabidopsis *(FDR-corrected alpha = 0.05). To examine broad-level classification of gene functions in the bracken transcriptome, we mapped GO terms to the GO-slim vocabulary (Figure 3) and repeated the Fisher's exact test. 42 GO-slim categories were overrepresented and 88 categories were underrepresented in the *Pteridium *transcriptome relative to the *Arabidopsis thalliana *GO-slim annotation (FDR-corrected alpha = 0.05; Additional file 3).

### Comparative genomics

**Figure 4:**
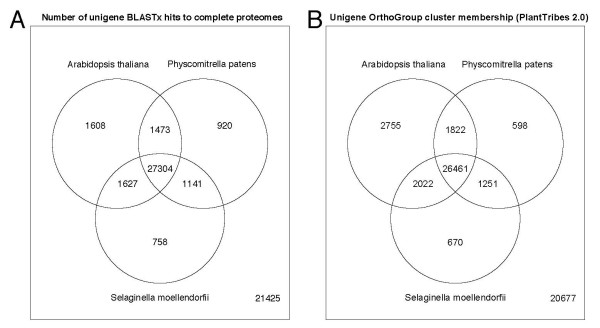
**Homologous gene detection in diverse plant proteomes**. (A) Blastx: The complete unigene set was queried against the complete set of predicted proteins in the genomes of *Arabidopsis thaliana*, *Physcomitrella patens*, and *Selaginella moellendorfii *using an e-value cut off of 1e-5. Unigenes with positive hits in more than one proteome are shown in the intersect for those species. Of 56,256 total unigenes, 21,425 unigenes did not have a positive blast hit. (B) PlantTribes OrthoGroups: Unigenes were assigned to Tribe- and OrthoMCL clusters derived from the updated PlantTribes classification based on the best blast hit for each unigene. The presence of genes from *Arabidopsis thaliana*, *Physcomitrella patens*, and *Selaginella moellendorfii *in each OrthoGroup was evaluated. Of 56,256 total unigenes, 18,368 unigenes were not assigned to an OrthoGroup cluster and an additional 2,309 were assigned to clusters having no homologs from *Arabidopsis*, *Physcomitrella*, or *Selaginella*.

**Figure 5:**
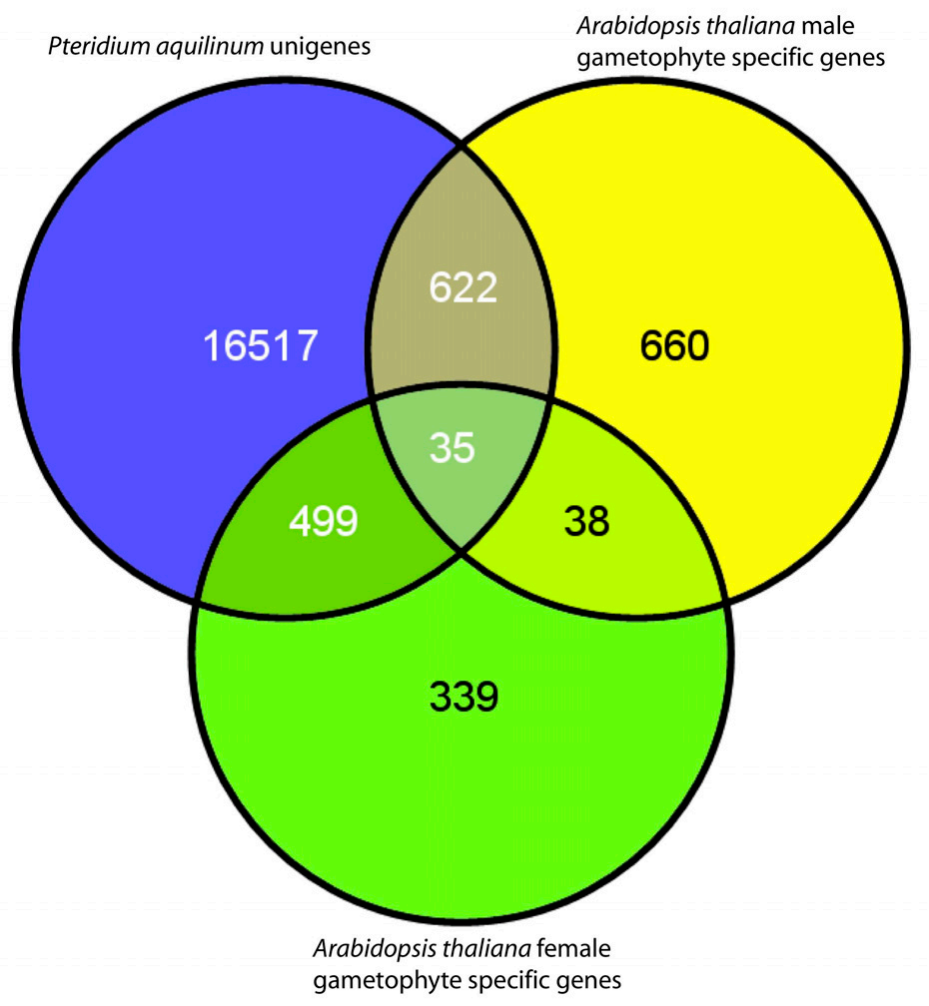
**Detection of homologs to *Arabidopsis *gametophyte genes**. To screen for the presence of potential gametophyte genes, *Arabidopsis *genes producing significant alignments with *Pteridium *unigenes in a blastx search (e-value cutoff = 1e-10) were compared with the list of male gametophyte specific and female gametophyte enriched genes identified from the literature [68-70].

Unigenes were classified into 6,987 tribe (inflation level 3) and 9,395 orthogroup MCL clusters (Additional file 4) in the PlantTribes gene family database on the basis of the best blastx hit to the inferred protein models of ten complete plant genomes included in an updated version of the PlantTribes database ([60] and CWD, unpublished). To evaluate the level of gene overlap between the *Pteridium *gametophyte transcriptome and other land plants, we examined overlap in both PlantTribes orthogroup cluster membership and blastx hits for predicted proteins in *Physcomitrella patens*, *Selaginella moellendorffii*, and *Arabidopsis thaliana *(Figure 4). Among genes in the *Arabidopsis *genome with positive blastx hits with *Pteridium *unigenes, we examined for the presence of "gametophyte genes" previously identified in the literature. Honys and Twell [68] used microarrays to identify 1,355 genes specifically expressed in haploid male gametophyte tissues in *Arabidopsis*, that is, genes consistently expressed in at least one of four male gametophyte developmental stages and absent in six sporophytic tissue gene expression profiles. Similarly, Yu et al. [69] and Wuest et al. [70] identified 911 genes (combined) that were significantly over-expressed in female gametophytic cells relative to sporophytic tissues. In total, we identified 1,156 known *Arabidopsis *gametophyte genes that produced significant alignments with *Pteridium *unigenes in our blastx search (Figure 5).

### Repetitive sequence characterization

A total of 2,679 perfect di-, tri-, tetra-, and pentanucleotide simple sequence repeats (SSRs) longer than 9, 8, 6, and 5 repeats, respectively, were identified in 2,285 unigenes (Additional file 5) using msatCommander [71]. Sufficient flanking sequences existed to design high quality primers for 548 potentially amplifiable SSR loci. PCR primers were chosen using Primer3 [72] as implemented in msatCommander [71] (Additional file 6). Since this RNA was extracted from gametophytes derived from spores collected from a single diploid sporophyte, we are unable to determine the level of variation present at these SSR loci in natural populations.

To identify and classify expressed repeat sequences, we screened the *Pteridium *unigenes with RepeatMasker, using RepBase sequences belonging to land plants (Embryophyta). In total, 416 retrotransposons were identified, representing 0.17% of the total unigene sequence length (Table 5). Additionally, 269 DNA transposons were identified, representing 0.07% of the total sequence length (Table 5).

**Table 5:** Repetitive transposon classification

Transposon class	Number of elements	Total length	Percentage of sequence
Retroelements	416	51,070	0.17%
LINE/L1	38	2,458	0.01%
LTR	378	48,612	0.16%
Copia	183	21,750	0.07%
Gypsy	195	26,862	0.09%
DNA transposons	269	22,699	0.07%
hobo-Activator	20	2,022	0.01%
Tc1-IS630-Pogo	1	46	0.00%
En-Spm	180	13,395	0.04%
MuDR-IS905	33	1,834	0.00%
Harbinger	6	368	0.00%
Rolling circle Helitrons	29	5,034	0.02%

Classification and frequency statistics for repetitive elements identified by RepeatMasker. The database used to screen unigenes was built with repeat sequences identified in RepBase as belonging to land plants (Embryophyta).

## Discussion

We have used high-throughput sequencing data to characterize the gametophyte transcriptome of *Pteridium aquilinum*, a species for which very little genomic data are available. These data represent an 865-fold increase over the expressed sequence data previously available for *Pteridium *in Genbank [9].

### Assembly quality

Because contaminant adapter/primer sequences, polyA/T repeats, and low complexity end sequences can substantially compromise *de novo *assembly and can be difficult to completely remove (KM Dlugosch, personal communication), we aggressively filtered and trimmed the reads beyond the default instrument-level processing routines at the cost of sequence information loss (approximately 11.6 Mbp were removed, representing 4.4% of the filter-passed data).

Considering the sheer quantity and depth of sequencing produced by next-generation sequencing platforms, we deemed this an acceptable level of loss to improve accuracy in the assembly. We also used a two-step assembly strategy to minimize redundancy in our final unigene sequence set. We adopted this approach because MIRA is able to handle the large number of reads produced by 454 sequencing and utilizes a multi-pass strategy to identify and correct sequencing and assembly errors to produce a highly accurate assembly, but is sensitive to uneven sequencing depth of coverage and allelic diversity, resulting in a high number of redundant contigs. CAP3 is a proven and efficient DNA sequence assembler that can be used to join highly similar overlapping sequences, but is unable to handle the large number of reads produced by new high-throughput sequencing platforms. By combining these two assembly tools, we were able to join contigs and singletons that failed to assemble in MIRA to reduce sequence-level redundancy in our final unigene sequence set.

In examining the taxonomic distribution of nr blastx hits for the unigenes, we identified only a small proportion of sequences with best blast hits or LCA assignments outside of the green plants. When we examine these hits in greater detail, we find that many of them only align to short conserved domains, are hypothetical proteins of unknown function from model organisms, or are genes which are conserved across broad taxonomic levels, such as cytochrome P450, alpha-tubulin and dynein proteins. Additionally, because no other fern genomes have been sequenced, some of these sequences may represent novel fern genes. Thus, the evidence indicates that there is very little heterospecific sequence contamination in these data.

### Transcriptome coverage

While the simulations that underlay ESTcalc are based on the well characterized *Arabidopsis thaliana *floral transcriptome (approximately 18,000 genes with transcripts averaging 1,500 bp long) and assume perfect cDNA normalization and sequence assembly, Wall et al. [57] show that their results were highly predictive for empirical datasets from diverse eukaryotic species and tissues, making their simulations useful as a null model for predicting transcriptome coverage in other organisms. The predictions for transcriptome coverage produced by ESTcalc are largely consistent with that observed in our two-step assembly. However, the larger assembly size, greater number of unigenes, and shorter unigene lengths observed in our data set relative to the ESTcalc prediction may be explained by imperfect cDNA normalization or inefficient *de novo *assembly. Additionally, it is also becoming evident that with increased transcriptome sequencing throughput, it is possible to capture a richer, more nuanced, picture of transcriptome complexity (e. g. partially processed transcripts and alternative splice forms) [73-75]. This increased information content, however, presents significant challenges for *de novo *assembly and often results in a fragmented or partially redundant assembly [76,77]. Also consistent with the ESTcalc estimate that we have tagged all of the transcripts present in this sample, our unigene accumulation curve shows that the rate of new unigene detection for this cDNA library has declined to the point that additional sequencing is unlikely to detect new genes, but may however serve to condense and join non-overlapping contigs in our assembly. Similar approaches to evaluate sufficient sampling in transcriptome projects have been used by other researchers when other information about the transcriptome is absent [15,78].

### Functional annotation

The GO functional categories represented in the *Pteridium *gametophyte transcriptome are not significantly different from the suite of functional categories present in the full Arabidopsis genome GO annotation. Most of the unigenes annotated with a cellular component are localized to plastids or mitochondria, but a large number of them are also targeted for ribosomes or the plasma membrane (Figure 3). The molecular function of unigenes is heavily dominated by binding nucleic acids or proteins and metabolic activity, including hydrolase and kinase activity (Figure 3). The biological processes represented include all of the major cellular processes from transport and cellular organization to transcription, translation, and metabolism (Figure 3).

Visual examination of annotated/colored KEGG maps (not shown) indicates that we have captured all of the genes required for glycolysis, the citrate cycle, and plant hormone biosynthesis including gibberellin, abscisic acid, strigolactone, cytokinin, brassinosteroid, and auxin. We also detected Enzyme code signatures for most of the genes involved in nucleic and amino acid metabolism and chlorophyll biosynthesis.

### Comparative genomics

The PlantTribes database contains an objective MCL cluster-based classification system for plant genes and gene families [60,79,80]. By identifying similar sequences in this classification system, we assigned unigene sequences with putative gene family identities. The most abundant of these gene families present in the unigene set was the pentatricopeptide repeat protein (PPR) family, with over 600 unigenes classified as PPR proteins. We were also able to identify 65 unigenes classified in the MADS-box transcription factor family. Using this classification, gene sequences from *Pteridium *can be extracted for gene families of interest for use in studies of gene family evolution or phylogenomics. The overlap in orthogroup membership and blast hits for proteins in *Arabidopsis thaliana*, *Selaginella moellendorffii*, and *Physcomitrella patens *is similar (Figure 4A/B), but some striking differences can be observed. In both the PlantTribes and blast-based Venn diagrams, most of the unigenes which were identified in *Arabidopsis*, *Selaginella*, or *Physcomitrella *are also shared across all three species. In the PlantTribes classification, most of the genes are shared with *Arabidopsis *(21,649 unigenes), the species in this comparison that shares the most recent common ancestor with *Pteridium*, while slightly fewer and approximately equal gene representation is shared with *Selaginella *and *Physcomitrella *(19,649 and 19,485 unigenes, respectively). This is in contrast to the blast-based examination of gene set overlap in which *Arabidopsis *again has the greatest number of unigenes with hits (23,148 unigenes), but *Physcomitrella *has hits with 6,122 more unigenes than *Selaginella *(Figure 4). At first this seems counterintuitive because *Pteridium *shares a more recent common ancestor with *Selaginella *than with *Physcomitrella*. This pattern may be explained by the expression of "gametophyte genes" in *Pteridium *that are conserved with genes in the *Physcomitrella *genome, however little is known about the expression and evolution of genes between sporophyte and gametophyte stages. Both *Physcomitrella *and *Pteridium *have maintained a homosporous life cycle with a large independent gametophyte stage. These life history differences may also play a role on the selective pressures and/or constraints influencing gene evolution and more work is needed to address these hypotheses.

In our examination of *Arabidopsis *gametophyte genes, we identified gametophyte-expressed homologs in *Pteridium *for over half (52.7%) of the previously characterized *Arabidopsis *gametophyte specific or enriched genes. This finding suggests that a highly specific suite of genes required for gamete production and syngamy may be conserved over long periods of evolutionary time, despite substantial differences in life cycle and reproductive strategies between *Pteridium *and *Arabidopsis*. It should be noted also that these conserved genes are not the genes required for meiosis because the tissues sampled in this study and those used to identify gametophyte specific genes in *Arabidopsis *were all post-meiotic. An in-depth study of sporophytic genes in *Pteridium *is needed to better understand the evolution and expression of genes between the sporophyte and gametophyte stages.

## Conclusions

This study is the first comprehensive sequencing effort and analysis of gene function in the transcriptome of a fern and represents the most extensive expressed sequence resource available in ferns to date, nearly 16 times more data than exists for *Adiantum capillus-veneris*. These data are an important new scientific resource for comparative evolutionary studies in land plants and will be of great value for studies of genome structure and function in ferns. These data can be used to develop microarrays for gene expression assays or serve as a reference transcriptome for future RNA-seq experiments in *Pteridium*. As additional genome-scale projects in diverse plants are undertaken, these data will be of immense value in representing ferns, the sister clade to seed plants, in comparative genomic analyses.

## Methods

### Gametophyte culture, library preparation, and sequencing

*Pteridium aquilinum *ssp. *aquilinum *spores (collection number: Wolf 83; sourced from a single sporophyte individual collected in Norwich, UK) were sown onto sterile agar nutrient media containing Bold's macronutrients and Nitch's micronutrients (prepared as described by [81]) and grown under white light. Whole gametophytes including both vegetative and sexually mature male, female, and hermaphroditic individuals of various ages (up to 9 months from germination) were flash frozen in liquid nitrogen and ground to a fine powder. Total RNA was isolated using the Sigma Spectrum Plant Total RNA Kit, incorporating on-column DNase I (Qiagen) digestion during extraction to remove traces of genomic DNA. Total RNA was concentrated by precipitating in 2.5 M ammonium acetate and 70% ethanol, then resolubilizing the RNA pellet in RNase-free water to approximately 500 ng/μL. Total RNA was quantified and its quality verified using an Agilent Bioanalyzer 2100. Total RNA was sent to the Center for Genomics and Bioinformatics at Indiana University, Bloomington (IU CGB), where a normalized transcriptome (cDNA) library optimized for Roche 454 GS-FLX Titanium sequencing was prepared [82].

Briefly, full-length enriched cDNA was synthesized with the CloneTech SMART cDNA synthesis kit using modified 454-ready adapter/primer oligos (K Mockaitis, unpublished). The frequency of abundant cDNA species was reduced using the Evrogen Direct Trimmer normalization kit. Normalized cDNA was fragmented by sonication, blunt end repaired, and ligated to custom 454 sequencing adapters. Amplification of the sequencing library incorporated an adapter-mediated PCR suppression effect to preferentially amplify ligation products suitable for 454 sequencing [17]. The final transcriptome library was size selected and 454 sequencing proceeded according to the manufacturers recommended protocol on 3 regions of a four region PicoTiter plate.

### Sequence preprocessing, transcriptome assembly, and coverage assessment

Sequence reads generated in this study were deposited in the NCBI sequence read archive (SRA012887). Raw sequence reads that passed instrument software quality filters were trimmed of custom oligonucleotide adapter sequences (Justin Choi, unpublished, IU CGB). The resulting sequences were further processed with SeqClean [83] and SnoWhite v1.0.3 [84] to remove low quality, short, and contaminant sequences, and to aggressively trim polyA/T sequences. Cleaned reads were assembled *de novo *in MIRA v3rc4 [55,85] using a minimum percent identity of 94% to align reads, retaining singleton reads in the assembly (-OUT:sssip = yes). This primary assembly was passed through a secondary assembly step in CAP3 (95% identity, 25 bp overlap) [56] to reduce redundancy in the final assembly and join additional contigs. Custom perl scripts were used to extract summary information about the reads and assemblies (Tables 1 and 2; Additional file 7).

We utilized a web-based tool, ESTcalc [57], to estimate the predicted level of transcriptome coverage for our data set. Input parameters to ESTcalc require that we specify the sequencing technology used (or a combination of technologies) and either the total sequencing level (Mbp), or the number of reads and an estimate of read lengths. We used the best approximation for sequencing technology available (454 GS-FLX) and the empirical values observed for the cleaned sequence data (254 Mbp or 681,722 reads with an average of 372.6 bp/read) to obtain our estimates. The estimates reported were identical whether we parameterized on total sequence or supplied read length information as well.

To determine the number of eukaryotic ultra conserved orthologs (UCOs [58]) we captured in the *Pteridium *transcriptome data set, we queried a list of 357 UCO coding sequences from *Arabidopsis *(sequences available at: http://compgenomics.ucdavis.edu/compositae_reference.php) into the unigene set with an e-value threshold of 1e-10 using NCBI tblastx. These blast results were then parsed to determine then number of UCOs with a positive hit that returned an amino acid alignment greater than 30 residues long.

We assessed the changing rate of new gene detection as a function of sampling effort (unigene accumulation curve, Figure 2) using a bootstrapped random sampling protocol implemented in a custom perl script (Additional file 8). This script uses the empirical distribution of read number per unigene in our final assembly to randomly sample reads one at a time and tracks the total number of unigenes detected at each step. Because the order of sampling can impact the shape of this curve, we computed 1,000 replicate random sample orders and calculated the mean number of unigenes detected after each draw. To evaluate the level of variation in the number of unigenes detected, we also calculated the 95% confidence interval on the number of unigenes. Using this curve, we then estimated the number of reads it took to capture an average of 90%, 95%, and 99% of the unigenes and the average number of additional reads required to detect each of the last 10 unigenes.

To evaluate for the presence of potential contaminating sequences, we examined the taxonomic distribution of blastx hits for the unigene set in the NCBI nr protein database using an e-value threshold of 1e-10. The top 10 blast hits for each unigene were kept and examined in MEGAN v.3.7.2 [61]. MEGAN is a tool built for the examination of metagenomic data sets and provides a number of useful functions to explore the information content of large blast results. The blast results for each unigene were mapped onto the NCBI taxonomy tree by examining just the best hit (lowest e-value) or by using the lowest common ancestor (LCA) algorithm [61]. LCA was determined using at least three blast hits with a bitscore greater than 75 and within 10% of the top bitscore for that unigene.

### Functional annotation

The same blast search used to examine the taxonomic distribution of blast hits was used to identify putative homologous proteins and annotate each sequence with gene ontology (GO) terms using Blast2GO [65-67]. Blast2GO was also used to automatically handle InterProScan (IPS) searches to identify conserved protein domains in translations of the longest ORF in each unigene. Any GO terms associated with IPS hits were then merged into the blast-based GO annotation. GO terms were then used to map enzyme codes to each sequence. Enzyme codes were then used to automatically color and retrieve KEGG pathway maps [86,87]. As a final step in examining a broad functional representation of the gametophyte transcriptome, GO terms were mapped to the reduced GO-slim ontology and visualized and explored with directed acyclic graphs (not shown) and summarized with filtered pie charts including GO categories represented by at least 150 sequences (Figure 3)

### Comparative genomics

Unigenes were classified into tribe- and orthoMCL clusters in the PlantTribes2.0 database using a custom Perl pipeline (dePamphilis lab, unpublished) which queries each unigene against the complete inferred protein set from ten plant species that have complete sequenced genomes, using a blastx e-value threshold of 1e-10. Unigenes were assigned to MCL clusters based on the best blast hit. Species used for blast searches and gene clustering in the PlantTribes2.0 database include: *Chlamydomonas reinhardtii v3.0*, *Physcomitrella patens v1.1*, *Selaginella moellendorffii v1.0*, *Oryza sativa v5.0*, *Sorghum bicolor v1.0*, *Vitis vinifera v1.0*, *Populus trichocarpa v1.0*, *Medicago truncatula v1.0*, *Carica papaya v1.0*, and *Arabidopsis thaliana *(TAIR7). Meta-information about each assigned cluster was extracted from the database for each unigene and was output to a file. Simple text-based searches examined this information to retrieve gene family names and putative gene family functional data. The shared single copy tribes [59] for *Arabidopsis*, *Vitis*, *Populus*, and *Oryza *were identified in the PlantTribes2.0 database and the number of these tribes detected in the unigene set was determined by examining the pipeline output file. Orthogroup assignments for *Pteridium *unigenes were examined for cluster membership by *Selaginella*, *Physcomitrella*, and *Arabidopsis *to generate a Venn diagram showing putative gene level overlap (Figure 4A). Unigenes were also directly queried against each of these proteomes using a blastx e-value threshold of 1e-10 to examine the distribution of similar proteins in these three species. Venn diagrams were generated to graphically illustrate the overlap of unigenes for each proteome (Figure 4B). To screen for the presence of putative gametophyte genes, a list of male gametophyte specific genes in *Arabidopsis *was extracted from the microarray study of Honys and Twell [68] and a combined list of significantly enriched female gametophyte genes was compiled from the studies of Wuest et al. [70] and Yu et al. [69]. These *Arabidopsis *gametophyte gene lists compiled from the literature were examined for overlap with the list of genes producing significant alignments with *Pteridium *unigenes in the blastx search against the *Arabidopsis *genome to produce a Venn diagram of gametophyte genes (Figure 5).

## Authors' contributions

JPD conceived and designed the study, cultured the plant tissue, isolated total RNA, performed the data analyses, and drafted the manuscript. MSB advised on sequencing strategies and assisted with bioinformatic analyses. CWD and NJW assisted with bioinformatic analyses, assisted in summarizing and interpreting analysis results and in planning the manuscript. PGW collected the original material used to initiate gametophyte tissue culture and provided input on all aspects of the study from experimental design and analyses to manuscript preparation. All authors have read and approved the manuscript.

## Supplementary Material

Additional file 1 **Unigene builds**. Unigene sequences in FASTA format, compressed zip file. Click here for file

Additional file 2 **Unigene functional annotations from Blast2GO**. GO and EC functional classification for unigenes. Click here for file

Additional file 3 **Functional annotation enrichment for GO-slim categories relative to the complete Arabidopsis genome**. FDR-corrected Fisher exact test for GO-slim categories represented in the *Pteridium *unigene set and the *Arabidopsis *genome. Click here for file

Additional file 4 **PlantTribes2.0 gene family classification**. Tribe and orthogroup assignments for each unigene and cluster membership for the ten proteomes included in PlantTribes2.0. Functional and gene family descriptors for clusters are primarily inherited from the *Arabidopsis thaliana *genes included in the cluster. Click here for file

Additional file 5 **SSR loci identified in msatCOMMANDER**. Repeat sequence information (repeat motif, location, and lenth) for SSR loci identified by msatCOMMANDER. Click here for file

Additional file 6 **Primer sequences and details for SSR loci**. Primer sequences for potentially amplifiable SSR loci selected using msatCOMMANDER. Click here for file

Additional file 7 **Secondary coverage perl script**. Script used to identify singleton reads and primary and secondary contigs and calculate assembly statistics. Click here for file

Additional file 8 **Unigene accumulation curve perl script**. Script to randomly select reads from the assembly and calculate the number of contigs detected to produce the unigene accumulation curve. Click here for file
